# Hospitalised Myocarditis and Pericarditis Cases in Germany Indicate a Higher Post-Vaccination Risk for Young People Mainly after COVID-19 Vaccination

**DOI:** 10.3390/jcm11206073

**Published:** 2022-10-14

**Authors:** Karsten Montag, Günter Kampf

**Affiliations:** 1 Independent Researcher, 16835 Lindow, Germany; 2 University Medicine Greifswald, 17475 Greifswald, Germany

## 1. Introduction

It was recently described that the overall risk of myopericarditis after receiving a COVID-19 vaccine is low, except for younger males receiving mRNA vaccines [1]. The meta-analysis was based on observational studies on people in the general population who had myopericarditis in temporal relation to receiving vaccines [1]. In order to determine if these findings were supported by the case numbers in Germany, we evaluated the number of hospitalised cases, including those receiving intensive-care treatment, with myocarditis or pericarditis before the pandemic (2019), during the pandemic but without COVID-19 vaccines (2020), and during the pandemic with COVID-19 vaccines (2021).

## 2. Materials and Methods

Hospital renumeration data from Germany based on the International Classification of Diseases (ICD-10) were analysed [2]. Myocarditis was assumed when the principal diagnosis was coded as I40.8 (other acute myocarditis), I40.9 (acute myocarditis, unspecified), or I51.4 (myocarditis, unspecified); pericarditis was assumed when the principal diagnosis was coded as I30.9 (acute pericarditis, unspecified). An adverse reaction to COVID-19 vaccines was assumed when the secondary diagnosis was coded as U12.9 (COVID-19 vaccines causing adverse effects in therapeutic use, unspecified). An adverse reaction to any vaccine including a COVID-19 vaccine was assumed when the secondary diagnosis was coded as Y59.9 (complications due to vaccines or biological substances) or T88.1 (other complications following immunisation, not classified elsewhere).

## 3. Results

In 2019 and 2020, there were no or only very few cases (<4) of myocarditis or pericarditis described as adverse events after any type of vaccination (Y59.9 or T88.1; Table 1). None of them required intensive-care treatment. In 2020, 32 cases of myocarditis or pericarditis were hospitalised as COVID-19 patients (U07.1), with 15 of them requiring intensive-care treatment. In 2021, the number of hospitalised myocarditis or pericarditis cases among juveniles (10–17 years) had increased from 270 (2019) and 196 (2020) to 506 (2021). In total, 11 cases (2.2%) were associated with COVID-19, 160 cases (31.6%) were associated with a COVID-19 vaccine or vaccination in general, and 32 cases required intensive-care treatment. Similar results were found for young adults (18–29 years).

## 4. Discussion

In 2019 and 2020, only very few cases of myocarditis or pericarditis were associated with vaccines, suggesting that only a few other vaccines contributed to the case number in 2021. The large increase in hospitalised myocarditis or pericarditis cases in 2021 compared to 2019 and 2020 is only partly explained by vaccine-related adverse events or COVID-19. A general underreporting of adverse events has been described previously and may be an explanation for this finding [3]. In addition, the vaccine’s efficacy for symptomatic COVID-19 decreased in Germany among juveniles aged 12 to 17 from approximately 90% in early December 2021 to approximately 10% in late April 2022. At the time, the protective effect for hospitalisation was mostly between 30% and 65%, with peaks up to 90% and 100%, based on overall low case numbers [4].

A recent study on the incidence of myocarditis in a cohort of 65,785 patients aged 18 to 39 years found an incidence of 9.1 cases per 100,000 booster doses up to 21 days after a booster vaccination. This rate was substantially higher than the 0.2 cases reported by the Vaccine Adverse Event Reporting System, which is a passive system relying on patients or providers to report events [5]. 

## 5. Conclusions

We support the call for a comprehensive and careful benefit–risk evaluation of COVID-19 vaccines.

## Figures and Tables

**Table 1:** Numbers of hospitalised cases in Germany between 2019 and 2021 with a principal diagnosis of myocarditis (ICD10 codes I40.8, I40.9, and I51.4) or pericarditis (I30.9) and a secondary diagnosis of COVID-19 (U07.1), an adverse event related to a COVID-19 vaccine (U12.9), or an adverse event related to any vaccine (Y59.9 and T88.1); source: InEK Datenportal [2].

	2019		2020			2021					
	Total	Thereof U12.9, Y59.9, or T88.1	Total	Thereof U12.9, Y59.9, or T88.1	Thereof U07.1	Total	Thereof U12.9	Thereof Y59.9 *	Thereof T88.1 **	Thereof U07.1	Total U12.9, Y59.9, or T88.1 ***
0– 9 years											
Hospital cases	55	0	37	0	4	38	0	0	0	4	0
Subgroup cases with intensive care	16	0	13	0	3	14	0	0	0	<4	0
10– 17 years											
Hospital cases	270	0	196	0	4	506	119	22	19	11	160
Subgroup cases with intensive care	73	0	62	0	4	117	28	<4	4	3	32
18– 29 years											
Hospital cases	1515	0	1.011	0	<4	1828	301	54	38	17	393
Subgroup cases with intensive care	370	0	316	0	0	499	91	17	7	5	115
30– 39 years											
Hospital cases	1049	0	745	0	7	1038	119	23	17	11	159
Subgroup cases with intensive care	252	0	196	0	3	230	28	6	7	4	41
40–49 years											
Hospital cases	773	<4	604	<4	3	737	45	8	6	8	59
Subgroup cases with intensive care	161	0	159	0	<4	159	7	<4	0	<4	7
50–59 years											
Hospital cases	900	<4	727	0	5	796	35	13	6	7	54
Subgroup cases with intensive care	199	0	185	0	<4	163	6	4	0	<4	10
60–79 years											
Hospital cases	998	<4	911	0	4	1069	33	5	6	14	44
Subgroup cases with intensive care	215	0	203	0	<4	178	6	<4	<4	5	6
≥80 years											
Hospital cases	174	0	205	0	3	233	3	<4	3	5	6
Subgroup cases with intensive care	38	0	48	0	0	50	<4	<4	0	<4	0
All age groups											
Hospital cases	5734	3	4436	<4	32	6245	655	123	85	77	863
Subgroup cases with intensive care	1324	0	1182	0	15	1410	167	33	11	24	211

* Number of cases with the additional secondary diagnosis U12.9 were subtracted; the value of the sum of individual age groups and value of the total of all age groups can differ due to unspecified values < 4 in individual age groups. ** Number of cases with the additional secondary diagnosis U12.9 or Y59.9 were subtracted; the value of the sum of individual age groups and value of the total of all age groups can differ due to unspecified values < 4 in individual age groups. *** Values “< 4” are not included in the total.

## Data Availability

The data supporting this article are available in InEK DatenBrowser at https://datenbrowser.inek.org/ (accessed 25 February 2022). Use of the data is restricted to non-commercial purposes.
